# Dynamics of Dopamine and Other Monoamines Content in Rat Brain after Single Low-Dose Carbon Nuclei Irradiation

**DOI:** 10.3390/life12091306

**Published:** 2022-08-25

**Authors:** Viktor S. Kokhan, Alexey A. Ustyugov, Vladimir A. Pikalov

**Affiliations:** 1 V.P. Serbsky Federal Medical Research Centre for Psychiatry and Narcology, 119034 Moscow, Russia; 2 Institute of Physiologically Active Compounds RAS, 142432 Chernogolovka, Russia; 3 Institute for High Energy Physics Named by A.A. Logunov of National Research Centre “Kurchatov Institute”, 142281 Protvino, Russia

**Keywords:** ionizing radiation, carbon nuclei, monoamines, dopamine turnover, norepinephrine, anxiety, spatial learning

## Abstract

Space radiation, presented primarily by high-charge and -energy particles (HZEs), has a substantial impact on the central nervous system (CNS) of astronauts. This impact, surprisingly, has not only negative but also positive effects on CNS functions. Despite the fact that the mechanisms of this effect have not yet been elucidated, several studies indicate a key role for monoaminergic networks underlying these effects. Here, we investigated the effects of acute irradiation with 450 MeV/n carbon (^12^C) nuclei at a dose of 0.14 Gy on Wistar rats; a state of anxiety was accessed using a light–dark box, spatial memory in a Morris water maze, and the dynamics of monoamine metabolism in several brain morphological structures using HPLC. No behavioral changes were observed. Irradiation led to the immediate suppression of dopamine turnover in the prefrontal cortex, hypothalamus, and striatum, while a decrease in the level of norepinephrine was detected in the amygdala. However, these effects were transient. The deferred effect of dopamine turnover increase was found in the hippocampus. These data underscore the ability of even low-dose ^12^C irradiation to affect monoaminergic networks. However, this impact is transient and is not accompanied by behavioral alterations.

## 1. Introduction

Space radiation, presented primarily by high-charge and -energy particles (HZEs), has a substantial impact on the central nervous system (CNS) of astronauts. HZEs are very deeply ionizing highly charged nuclei with an atomic number of more than two. An important HZE parameter is linear energy transfer (LET)—the rate of particle energy loss, measured in keV/µm of water. During interplanetary missions, the vast majority of collisions will be produced by low-LET particles (<15 keV/µm), while the number of collisions and, accordingly, the absorbed dose for high-LET particles will be relatively small [1,2]. ^12^C nuclei are the most encountered after alpha particles among the nuclei flux in interplanetary space and the most encountered among HZEs [3]. Despite the obvious negative effect of HZEs on CNS [4,5], studies on the latest modern ground-based models that most appropriately reproduce outer space conditions have shown rather unexpected results. Among these findings was the recently discovered phenomenon of the composite effect of radiation. Combined exposure did not cause any cognitive impairments [6,7] that have been shown recently at comparable space-related doses (<1 Sv) under exposure to only one nuclei beam [8,9,10]. Moreover, combined irradiation revealed a pro-cognitive (^12^C, 0.14 Gy, 450 MeV, 10.3 keV/µm, and 0.4 Gy γ-rays) [11,12] and nootropic (^12^C 0.18 Gy, 450 MeV, 10.3 keV/μm, and γ-rays 0.24 Gy) [13] effect. The molecular mechanisms of these phenomena remain poorly understood, and the exact pathways that are involved in the composite action of ionizing radiation, including HZEs, remain unknown. At the same time, the growing body of research indicates that monoaminergic neural networks could be involved and serve as a key link in the implementation of the described phenomena [14,15].

One of the first pieces of evidence of functional alterations of the dopaminergic system was the detection of cocaine-induced locomotor response impairment after irradiation with ^56^Fe (1.2 or 2.4 Gy, 1 GeV, 147 keV/µm) with dose-dependent persistence in time [16]. It has been shown in two independent studies using H^+^ (150 MeV, 0.4 keV/µm) that irradiation caused a significant decrease (0.5 Gy, including non-sensitive to radiation animals) or increase (1 or 2 Gy) in dopamine (DA) transporter (DAT) levels and also increased (1 Gy) the level of DA receptor D_2_ in a brain slice (which included the caudate–putamen, nucleus accumbens, ventral tegmental area, and substantia nigra) and in the frontal cortex (only DAT). These observations were made in a cohort of irradiated rats only with behavioral impairments, and this group was designated as “sensitive to radiation” [17,18].

On the other hand, the overexpression of D_2_ receptors in the striatum after combined exposure to radiation (combined H^+^, 1.5 Gy, 170 MeV, 0.4 keV/µm, and 3 Gy γ-rays) and antiorthostatic suspension (a simulated model of microgravity) have suggested a neuroreparative effect of irradiation [19]. The analysis of serotonin metabolism has shown that irradiation suppressed serotonin turnover in the prefrontal cortex and hippocampus [15,19,20,21,22,23] but activated serotonin turnover in the hypothalamus [15]. The overexpression and increased protein level of 5-HT_2a_ receptors under combined irradiation (combined H^+^ 1.5 Gy, 170 MeV, 0.4 keV/µm, and 3 Gy γ-rays or combined ^12^C, 0.14 Gy, 450 MeV, 10.3 keV/µm, and 0.4 Gy γ-rays) have revealed pro-cognitive effects [19,24].

Despite the generally positive research data for future space missions, there is still a great need for fundamental comprehensive research on the influence of ionizing radiation, particularly HZEs, on biological subjects. A number of positive effects of the combined irradiation with γ-rays and ^12^C nuclei on the CNS and their connections with monoamine turnover and metabolism have been identified, but the effects of ^12^C beam irradiation in comparative doses are not yet thoroughly characterized.

To address these knowledge gaps, here, we define how ^12^C irradiation of the head affects behavior and monoamine metabolism and dynamics in male Wistar rats. The irradiation model used in the study is not relevant to interplanetary flights but is designed to facilitate an understanding of the interaction nature of γ-rays and ^12^C nuclei when combined.

## 2. Materials and Methods

### 2.1. Animals

Male 3-month-old Wistar rats weighing 270–300 g were used in this study. The rats were maintained at the Center for Collective Use of the Institute of Physiologically Active Compounds RAS (Chernogolovka, Russia). Rats were housed in a standard environment (12 h light/dark cycle, 18–23 °C, and 30–70% relative humidity) with food and water ad libitum.

Experimental groups were formed by random assignment of rats to: control animals (C, *n* = 16) and irradiated rats (R, *n* = 16). At Day 1 after irradiation, half of the control (C1, *n* = 8) and irradiated (R1, *n* = 8) rats were euthanized for the HPLC study. The remaining animals were subjected to a battery of behavioral tests and euthanized on Day 25 after irradiation: control (C25, *n* = 8) and irradiated (R25, *n* = 8) groups.

The study was approved by the Ethics Committee of Institute of Physiologically Active Compounds (protocol code 55 on 26 March 2021).

### 2.2. Carbon (^12^C) Nuclei Irradiation

The heads of experimental animals (R group) were subjected to a single exposure of ^12^C nuclei (450 MeV/*n*, 140 ± 15 mGy, LET 10.3 keV/μm) in a U-70 accelerator (NRC “Kurchatov Institute”—IHEP, Protvino, Russia). Dosimetric monitoring of irradiation was performed using a DKS-AT5350/1 dosimeter (Atomtex, Minsk, Belarus) with a TM30010-1 ionization chamber (PTW-Freiburg, Freiburg, Germany). A collimator from carbon (density 1.8 g/cm^3^, thickness 50 cm, diameter of beam 65 mm) was used to restrict the ^12^C beam. The unevenness of the irradiation field was <±2.5% within the sweep radius R = 30 mm. The animals were placed in special poly(methyl methacrylate) cases that restrict movement and were positioned so that only the head and a small area of the neck fell into the ^12^C beam.

The control animal group (C1 and C25 groups) was transported together with irradiated animals and also placed in cases but was kept separately in another laboratory room and, thus, not exposed to ionizing radiation.

### 2.3. Dark–Light Box

A dark–light box (OpenScience, Moscow, Russia) for rats was used where the light compartment was evenly lit (60 lx). Each rat was placed in the dark compartment, and movement activity indicators were recorded for 5 min.

### 2.4. Morris Water Maze

For these experiments, a black pool 180 cm in diameter was used. A black plastic platform (invisible to the animal) 10 cm in diameter was immersed 2 cm under water, always in the same place. The rat was released into the water at the edge of the pool at a chosen point and allowed to swim freely for 60 s. The same starting point was used for all rats within one trial. There were four trials per day, each time changing the starting point. The probe test was performed after the final learning at Day 6 of learning. The platform was removed from the pool, the rat was released into the water’s edge, and swim time to find the platform in the same quadrant as the platform was recorded within 60 s of total swim time.

### 2.5. Tissue Collection and High-Performance Liquid Chromatography

On Day 1 and Day 25 after irradiation, the appropriate group of rats were euthanized by decapitation, and the following brain morphological structures were isolated on a thermoelectric surface (+2 °C) and frozen in liquid nitrogen: the prefrontal cortex (PFC), hippocampus (HPC), striatum (ST), amygdala (AMY), and hypothalamus (HYP).

Brain tissue homogenization and extraction were carried out in 0.1 M HClO_4_ solution with the addition of 0.25 nM/mL 3,4-dioxybensilamine as an internal standard. Samples were centrifuged at 10,000× *g* for 15 min 4 °C. The content of monoamines (NA, 5-HT, DA) and their metabolites 5-hidroxyindoacetic acid (5-HIAA), homovanillic acid (HVA), 3,4-dihydroxyphenylacetic acid (DOPAC), and 3-methoxytyramine (3-MT) was determined in the supernatant using high-performance liquid chromatography (ion-pair chromatography) with electrochemical detection on a System Gold liquid chromatograph (Beckman Coulter, Inc., Brea, CA, USA) equipped with a Rheodyne 7125 injector (Rohnert Park, CA, USA) with a 20 μL sample loop. The samples were separated on a reverse-phase column Nucleodur C18 Gravity, 4.6 × 250 mm, pore diameter of 5 μm (Mashery-Nagel GmbH & Co., KG, Düren, Germany). Mobile phase (0.1 M citrate-phosphate buffer pH 3.0 containing 1.1 mM octanesulfonic acid, 0.1 mM EDTA, and 9% acetonitrile) flow rate of 1 mL/min at a pressure of 200 atm was achieved using a System Gold 125 pump (Beckman Coulter, Inc.). Measurements were performed using an EC3000 electrochemical detector (RECIPE Chemicals + Instruments GmbH, Munich, Germany) equipped with a ClinLab ECD cell, Model Sputnik, with a glassy carbon working electrode (+0.85 V) and a Ag/AgCl reference electrode. Sample registration and chromatogram processing were performed using Multichrom v.1.5 software (Ampersand, Moscow, Russia).

### 2.6. Data Processing

Data are presented as the mean ± standard deviation (SD). Standard data processing was performed using Statistica 12 software (StatSoft Inc., Tulsa, OK, USA). The Shapiro–Wilk test was used to assess the normality of the data distribution; for *p* > 0.05, parametric analysis methods were used. Intergroup differences in dark–light box were analyzed using Student’s *t*-test. Intergroup differences in Morris water maze were analyzed using repeated-measures analysis of variance (ANOVA). HPLC data were processed using two-way ANOVA. Duncan’s posterior test was calculated if necessary.

## 3. Results

### 3.1. Irradiation Does Not Change Trait Anxiety

**Figure 1:**
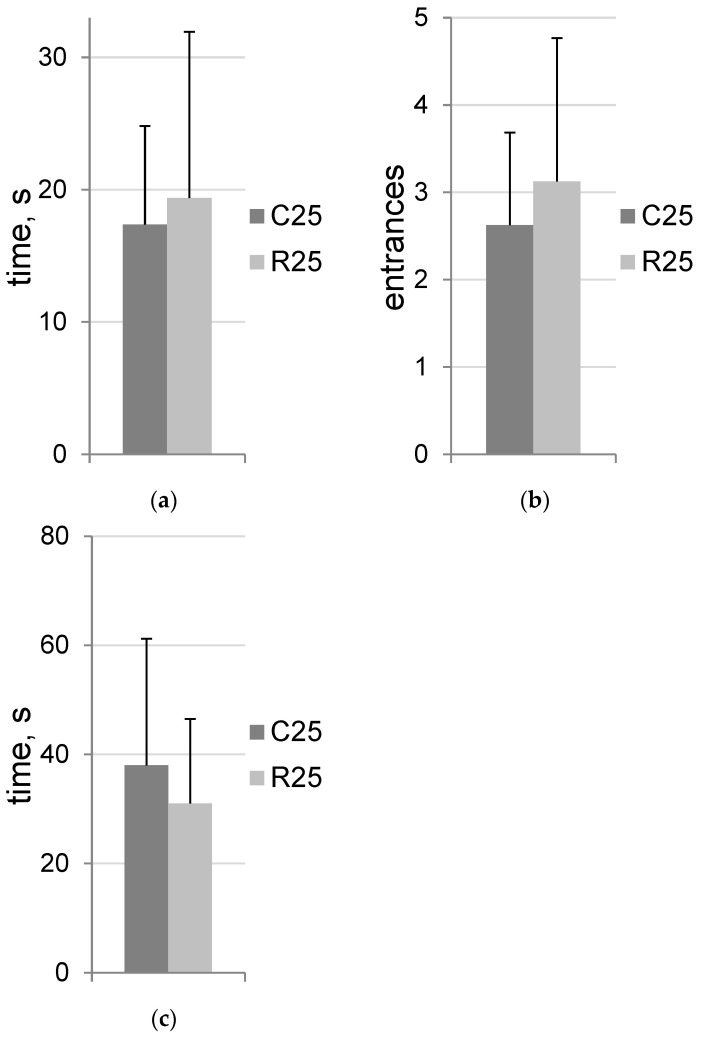
Dark–light box. (**a**) Latency time of light compartment entrance; (**b**) number of entrances; (**c**) total time spent in the light compartment. C25 (*n* = 8)—control group of rats; R25 (*n* = 8)—irradiated group of rats on Day 25 after exposure.

The dark–light box data are presented in Figure 1. No statistically significant differences were detected. All tested groups showed rodent-specific exploratory behavior without signs of increased state anxiety.

### 3.2. Irradiation Does Not Affect Spatial Training

**Figure 2:**
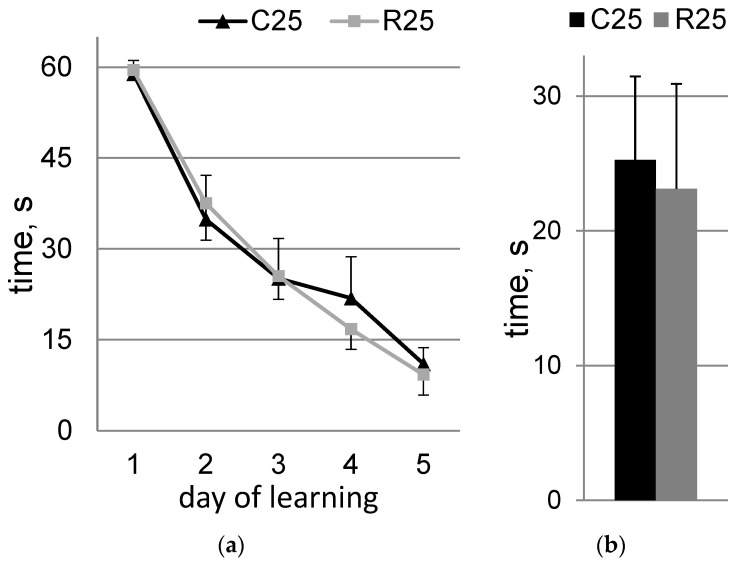
Morris water maze. (**a**) Learning dynamics; (**b**) probe test. C25 (*n* = 8)—control group of rats; R25 (*n* = 8)—irradiated group of rats on Day 25 after exposure.

The Morris water maze data are presented in Figure 2. No statistically significant differences were detected, both in the dynamics of learning (Figure 2a) and during the probe test (Figure 2b). Experimental groups of rats were characterized by good learning dynamics.

### 3.3. Irradiation Leads to Immediate and Transient Changes in Monoamine Metabolism, with the Exception of Hippocampus, Where It Appears Later

**Figure 3:**
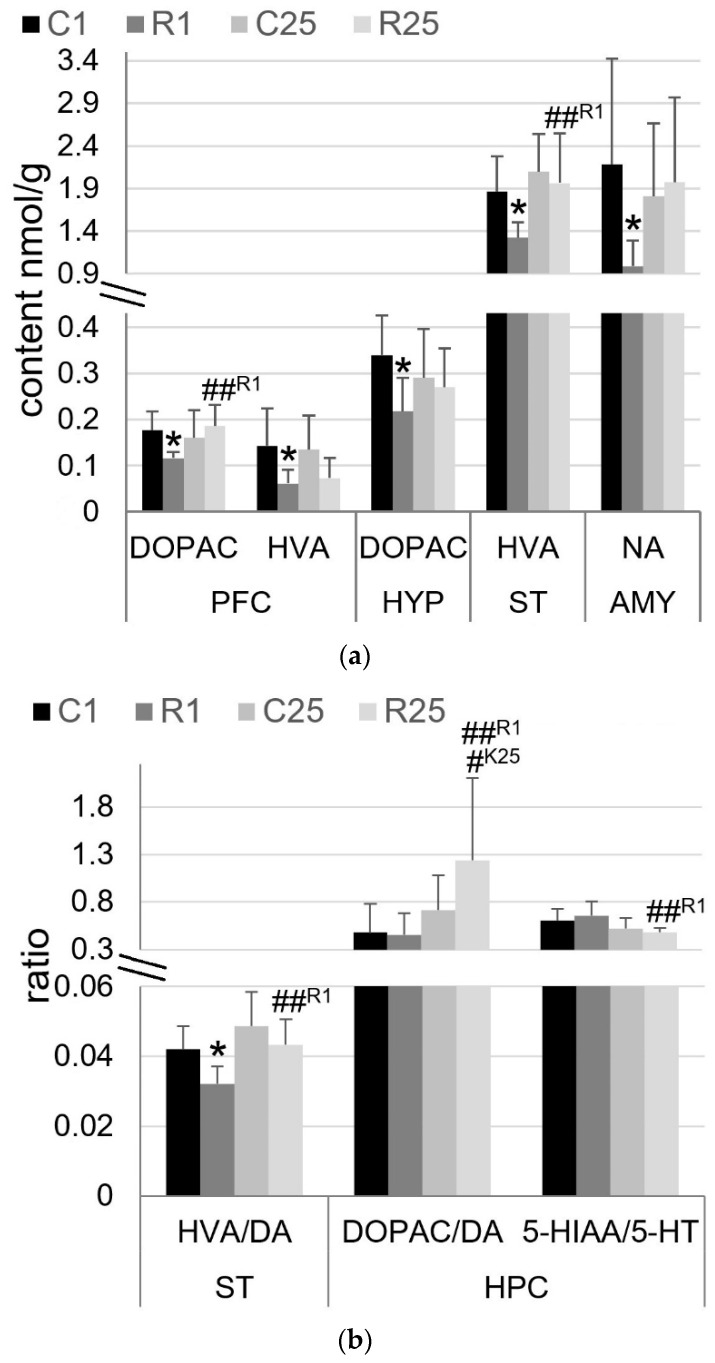
Content of monoamines and metabolites. (**a**) Absolute values of monoamine content, nmol/g; (**b**) values of metabolite/monoamine ratios. DA—dopamine, 5-HT—serotonin, NA—norepinephrine, 5-HIAA—5-hydroxyindoleacetic acid, DOPAC—3,4-dihydroxyphenylacetic acid, HVA—homovanillic acid, AMY—amygdala, HPC—hippocampus, HYP—hypothalamus, PFC—prefrontal cortex, ST—striatum. C1 (*n* = 8) and R1 (*n* = 8)—control and irradiated group of rats on Day 1 after exposure, respectively; C25 (*n* = 8) and R25 (*n* = 8)—control and irradiated group of rats on Day 25 after exposure, respectively. Asterisks (*) indicate statistically significant differences between K1 and R1 group of rats (* *p* < 0.05; post hoc Duncan’s test). Hash (#) indicates statistically significant differences between experimental groups except the K1 group of rats (# *p* < 0.05; ## *p* < 0.01; post hoc Duncan’s test).

The following statistically significant differences in monoamines and their metabolites were found in PFC (Figure 3a): DOPAC (F_1,28_ = 7.8, *p* = 0.009; radiation × time factor interaction) and HVA (F_1,28_ = 11.1, *p* = 0.002; time factor). The irradiated R1 group of rats showed a reduction in the monoamines DOPAC and HVA, at 34% (*p* = 0.011) and 57% (*p* = 0.019), respectively, in comparison with control C1 rats. In a separate group of irradiated rats—R25—DOPAC content was 60% (*p* = 0.005) higher than that in R1-rats.

Statistically significant differences in the following monoamines and their metabolites were found in HPC (Figure 3b): DOPAC/DA ratio (F_1,28_ = 8.1, *p* = 0.008; time factor) and 5-HIAA/5-HT ratio (F_1,28_ = 10.4, *p* = 0.003; time factor). The DOPAC/DA ratio was greater in the R25 group of irradiated rats: 73% (*p* = 0.049) compared to the value of control C25-rats and 172% (*p* = 0.008) compared to the value of R1-rats. In contrast, the 5-HIAA/5-HT ratio was decreased in R25-rats at 26% (*p* = 0.008) compared to that seen in R1-rats.

Statistically significant differences in the following monoamines and their metabolites were found in ST (Figure 3a,b): HVA (F_1,28_ = 4.9, *p* = 0.003, radiation factor; F_1,28_ = 8.3, *p* = 0.008, time factor) and HVA/DA ratio (F_1,28_ = 8.6, *p* = 0.007, radiation factor; F_1,28_ = 12, *p* = 0.002, time factor). In the R1 group of rats, decreased HVA content was found with mean HVA/DA ratios at 29% (*p* = 0.018) and 24% (*p* = 0.012) in comparison with C1-rats. In the R25 group of irradiated rats, the HVA content and mean HVA/DA ratio were 48% (*p* = 0.007) and 35% (*p* = 0.006) higher than those in R1-rats.

Statistically significant differences in the following monoamine and its metabolites were found in AMY (Figure 3a): NA (F_1,28_ = 4.3, *p* = 0.046; radiation × time factors interaction). The NA content in the R1 group was 55% (*p* = 0.023) compared with the C1 control group.

Statistically significant differences in the following monoamine and its metabolites were found in HYP (Figure 3a): DOPAC (F_1,28_ = 5.2, *p* = 0.03; radiation factor). The DOPAC content was lower in the R1 group at 36% (*p* = 0.016) compared to the value seen in C1 control rats.

## 4. Discussion

The importance of studying anxiety behavior is based on a number of data showing the relationship between radiation-induced anxiety and changes in the cognitive abilities of experimental animals. Thus, radiation-induced anxiety-associated cognition enhancement [15,25], decline [26,27], and the absence of changes against the background of anxiolytic drugs [28] were shown. The anxiogenic effect of ionizing radiation has been revealed in a number of relevant irradiation scenarios: γ-rays in a dose range of 0.5–2 Gy [8], combined irradiation (0.4 Gy γ-rays and ^12^C 0.14 Gy, 10.3 keV/μm) [15,25], and chronic exposure to neutrons and γ-rays (^252^Cf, 1 mGy/day, 0.4 Gy totally, but not ~0.12 or 0.2 Gy) [12].

During behavioral testing, no anxiogenic action of irradiation was detected. The decrease in NE content in AMY demonstrated an overall neurochemical picture indicating the absence of any anxiogenic effect caused by irradiation. Indeed, the reduced efficiency of NE innervation of AMY leads to an anxiolytic effect [29,30], whereas activation, conversely, is accompanied by the enhancement of signs of anxiety-like behavior [31,32]. At the same time, reduced NE content in AMY may be responsible for memory impairment in behavior tests that are based on electric foot shock and other aversive stimuli [33,34,35].

We found no impairment, and more importantly, no enhancement in the spatial learning of irradiated rats. The absence of learning disabilities in the Morris water maze is not surprising since a number of studies have not previously revealed any impairments in this test in close dose/nature of irradiation [36,37,38]. At the same time, the enhancement of spatial learning is detected at the same radiation doses of ^12^C irradiation under the condition of γ-ray (0.4 Gy) pre-irradiation [15].

Thus, the lack of anxiogenic action and spatial memory changes distinguishes acute ^12^C irradiation from previously observed data. Relying on combined irradiation data [15,25,28], we believe that the irradiation caused by the γ-ray component in combined irradiation conditions is key to inducing anxiogenic effects and cognition changes.

The analysis of the content of monoamines revealed that acute irradiation led to the immediate suppression of DA turnover in PFC, ST, and HYP. This conclusion is supported by the fact that an increased concentration of metabolites in the tissue ex vivo and/or elevated metabolite/neurotransmitter ratio represents increased neurotransmitter signaling in vivo [39,40]. However, on Day 25 after irradiation, these changes were no longer detected. The suppression of DA turnover in PFC and ST is also characteristic of acute irradiation with high-LET H^+^ (1 and 2 Gy, spread-out Bragg peak) [41] but not by low-LET H^+^ (1.5 and 3 Gy, 165 MeV) [20]. Taking into account the results of previous studies, we hypothesize that the suppression of DA turnover is dose-dependent in nature: from the transient in the present study to persistent (it immediately appears and lasts up to 30 but not 90 days after irradiation) when irradiated by ^12^C (500 MeV) in the absorbed dose 1 Gy, which was shown previously [21,23]. The decreasing content of DOPAC and HVA, first of all in PFC and HYP, may be associated with a decrease in the content or activity of monoamine oxidases but not catechol-O-methyltransferase. This assumption finds its confirmation in the absence of changes in the 3-MT content [42]. On the contrary, large doses of (1-3 Gy) low- and high-LET H^+^ led to significant changes in the content of 3-MT but not DOPAC [20,41], and in close conditions of irradiation, a change in the expression of catechol-O-methyltransferase was shown [19], which indirectly confirms our assumption. The change in the expression level of monoamine oxidase A is a similar indirect confirmation of our assumption [28]. However, the previous data [28] were obtained in several other conditions of irradiation (combined γ-ray and ^12^C) and on the background of anxiolytic pharmaceutical therapy; therefore, they must be considered with due caution.

The suppression of DA turnover in PFC, ST, and HYP may indicate a neurotoxic effect as a result of biomolecular damage, oxidative stress, and neuroinflammation caused by irradiation [43,44,45]. On the contrary, the increase in DA turnover in HPC could be considered as pro-cognitive and a sign of neurorecovery [46,47,48]. However, these assumptions are speculative and require future precision studies. Moreover, the observed neurochemical changes did not reach the degree in which CNS functional disorders would be observed in behavioral tests.

It is known that irradiation with γ-rays (fractionated 0.5 Gy × 6 times, 661.7 keV, and 3 Gy) does not affect monoamine turnover [49] or cognition (2 Gy acute or fractions 0.2 Gy × 10 days) [50]. Thus, we hypothesize that pre-irradiation with γ-rays before HZE irradiation produces unique effects that are not based on synergism or simple summation of individual effects. Moreover, γ-ray pre-irradiation, when combined in the irradiation model used, caused protective/recovery effects in the CNS [15,25]. A similar effect is probably observed when irradiation involves ultra-low-LET particle components, such as H^+^ or ^4^He, in combination with low-, moderate-, and high-LET nuclei in space-related doses (about 1 Sv) [6,7]. Additionally, the modulation of monoaminergic neural networks under irradiation makes a significant contribution to the exercise of these effects.

It is important to note that the doses used in the presented study are relevant neither to ionizing radiation during deep space missions nor to the therapeutic beam in radiotherapy. The main purpose of this work was to show the cardinal difference between mono-beam HZE irradiation and irradiation using pre-irradiation with ultra-low-LET sources (γ-rays in our previous study). At the same time, in the comparative analysis, we relied on a number of data from combined irradiation (γ-rays and ^12^C) obtained earlier [15,25,28].

## Data Availability

The data presented in this study are openly available in Mendeley Data, link Kokhan, Viktor (2021), “Dynamics of dopamine and other monoamines content in some rat brain morphological structures after single low-dose carbon nuclei irradiation”, Mendeley Data, V1, doi:10.17632/xn3z368d4t.1.
